# Taxonomic and functional diversity of the microbiome associated with the freshwater sponge *Metania* sp. (*Haplosclerida*: *Metaniidae*) from the Brazilian Cerrado, a metagenomic approach

**DOI:** 10.1186/s12866-026-05301-3

**Published:** 2026-06-20

**Authors:** Carla Patrícia Pereira Alves, Otávio Henrique Bezerra Pinto, Georgios Joannis Pappas, Sula Salani Mota, Janina Rahlff, Ricardo Henrique Krüger

**Affiliations:** 1 Molecular Biotechnology Centre, Universidade de Brasília (UnB), Brasília, 70910-900 Brazil; 2 Genomics for Climate Change Research Center, Universidade Estadual de Campinas, Campinas, 13083-875 SP Brazil; 3 Centro de Biologia Molecular e Engenharia Genética, Universidade Estadual de Campinas, Campinas, 13083-875 SP Brazil; 4 Department of Cell Biology, Universidade de Brasília, Brasília, 70910-900 DF Brazil; 5 Universidade do Distrito Federal (UnDF) Professor Amaury Maia Nunes, Brasília, DF Brazil; 6 Leibniz Institute on Aging - Fritz Lipmann Institute (FLI), Jena, Germany; 7 Department of Biology and Environmental Science, Centre for Ecology and Evolution in Microbial Model Systems (EEMiS), Linnaeus University, Kalmar, Sweden; 8 Aero-Aquatic Virus Research Group, Faculty of Mathematics and Computer Science, Friedrich Schiller University Jena, Jena, Germany

**Keywords:** Holobiont, Metazoans, Symbiosis, Metagenome

## Abstract

**Background:**

Sponges, the oldest metazoans on the planet, have an evolutionary history shaped by symbiotic associations with microorganisms. Although well studied in marine sponges, these associations are poorly understood in freshwater species. This study explored the taxonomic diversity and functional potential of the microbiome of the freshwater sponge *Metania* sp. and its distinction from the surrounding water, using a metagenomic approach. The samples were collected in the Brazilian Cerrado.

**Results:**

Taxonomic assignment identified 17 phyla, including bacterial and archaeal, with 19 sequence variants successfully assigned to the species level. Bacteria comprised 16 phyla, with a predominance of *Pseudomonadota*, *Actinomycetota*, and *Bacteroidota* in both microbiomes. The sponge microbiome is distinct from the water microbiome (PERMANOVA; F = 21.6, *p* = 0.04), sharing only 27% of the identified taxa. Functional prediction resulted in 7,201 KEGG Orthologs (KOs), assigned to 117 significantly enriched metabolic pathways. Although 95 pathways are shared, differential abundance analysis identified 1,024 KOs more abundant in the sponge microbiome and 1,275 in the water. The presence of bacterial defense systems such as CRISPR-Cas in the sponge microbiome suggests a crucial role in protecting against phages while maintaining symbiosis. In contrast, the water microbiota is enriched with pathways linked to environmental adaptation, such as secondary metabolite biosynthesis and pollutant degradation. Although the water microbiome harbored 1.3 times more biosynthetic gene clusters (BGCs), the sponge microbiome also demonstrated biotechnological potential for producing secondary metabolites, especially antimicrobial.

**Conclusions:**

These findings demonstrate that the freshwater sponge *Metania* sp. hosts a complex and functionally specialized microbial community that plays fundamental roles in adaptation, nutrition, and defense, highlighting the critical importance of symbiotic associations for the host.

**Supplementary Information:**

The online version contains supplementary material available at 10.1186/s12866-026-05301-3.

## Background

 Sponges are metazoans belonging to the phylum *Porifera*. They are sessile, filter-feeding aquatic organisms that directly contribute to water purification, the cycling of organic matter, and the energy balance of aquatic ecosystems. Despite their simple morphology, they establish complex ecological interactions with other organisms, acting as true ecosystem engineers [1, 2]. In addition, they are currently considered one of the most promising sources of bioactive compounds [1, 3]. Their lifestyle and evolutionary history certainly converge in survival strategies where an efficient innate immune system and the synthesis of secondary metabolites are functionally integrated [4]. These metabolites act as chemical effectors of the sponge’s immunity, providing a primary defense mechanism against pathogens and competitors [4, 5]. Secondary metabolites produced by sponges, such as alkaloids and terpenes, have pharmacological applications, including antitumor, anticancer, and anti-infective activities, as well as uses in cosmetics [5, 6].

*Metania* Gray, 1867 is the type genus of the family *Metaniidae* (*Spongillina*: *Haplosclerida*), which comprises five genera. Currently, 11 species of *Metania* are described and accepted, presenting circumtropical distribution in the Neotropical, Afrotropical, Oriental and Australian regions [7, 8]. The five species from the Neotropical region are found in Brazil (*M. fittkaui*, *M. kiliani*, *M. reticulata*, *M. subitilis*, and *M. spinata*), with *M. reticulata* and *M. spinata* being the most common [8]. The genus is characterized by unique spined megascleres (acanthoxeas) and specialized gemmular structures that distinguish it from other freshwater sponges [7, 8]. Previous research has broadened our understanding of seasonal life cycles [9, 10], environmental drivers of spicule production [11], and chemical compounds [12, 13]. However, significant gaps remain in molecular phylogenetics and symbiotic relationships with microorganisms. Although there are records of microorganism diversity associated with gemmules of Amazonian sponge *M. reticulata* [14] and studies of bioactive components produced by bacteria isolated from this species [15], substantial deficiencies persist in the realms of molecular phylogenetics and the symbiotic interactions with microorganisms.

Symbiosis with microorganisms has an evolutionary basis, with species-specific assemblages, mainly bacteria such as *Pseudomonadota*, *Actinomycetota,* and *Bacteroidota*, that are distinct from those in the surrounding environment [16–19]. Although marine sponges have been extensively studied, freshwater species still represent an unexplored frontier regarding their microbial associations [20]. Currently, of the 240 freshwater sponge species described worldwide, less than 10% have studies focused on their symbiotic bacterial communities [20]. The microbial compositions of several freshwater sponges (*Spongilla lacustris*, *Corvospongilla lapidosa*, *Ephydatia fluviatilis*, *E. muelleri*, and *Eunapius carteri*) have been characterized using several approaches, including cultivation-dependent and molecular techniques [21–26]. Similarly, endemic species from Lake Baikal, such as *Lubomirskia baikalensis* and *Baikalospongia* sp., have undergone extensive investigation, extending to the microbial profiles of diseased individuals [27–29].

Research in this area, published between 2005 at 2023, has progressed from early molecular characterizations based on clone libraries [22–24] to modern metagenomic approaches [21, 25, 29] and functional analyses of biosynthetic genes [27, 30, 31]. The main contributions of these studies are that freshwater sponges harbor distinct and diverse bacterial communities, dominated by *Pseudomonadota* (*Proteobacteria*), *Bacteroidota* (*Bacteroidetes*), and *Actinomycetota* (*Actinobacteria*), with evidence to host selection, vertical transmission and significantly biosynthetic potential. Findings include the identification of novel bacterial lineages, broad antimicrobial activities, unique polyketide synthase (PKS) genes, and associations between microbial imbalances and mass mortality events. Recent studies highlight the role of environmental factors in structuring sponge microbiomes, such as water quality, pollutants, and seasonality [32, 33] .

Symbiotic microorganisms perform crucial functions such as contributing to host nutrition, producing secondary metabolites with biotechnological potential, and protecting against pathogens [34–38]. The characterization of microbial communities, previously limited to cultivation methods, has been revolutionized by molecular techniques. Shotgun sequencing of the metagenomic DNA and computational tools have emerged as powerful instruments, enabling the rapid and cost-effective sequencing [39] of high-quality draft microbial genomes (> 90% completeness and < 5% contamination) [40]. This innovative approach allows for a comprehensive understanding of microbial diversity, revealing complex interactions, metabolic pathways, and ecological functions in diverse environments [41].

Most of investigations about microbial symbiosis with freshwater sponges were conducted on species collected on the European continent (Russia, Germany, Norway, and the Netherlands) [21–24, 29–31], India [25], and the United States [33, 42]. Among the freshwater sponge species studied for their associated microbiota, three species have been collected in Brazil: *Tubella variabilis* in Pernambuco [43] and *Metania reticulata* and *Heteromeyenia cristalina* in Amazonas [14, 15]. The central region of Brazil remains largely unexplored in terms of sponge diversity and associated microbiomes.

The Central Plateau of Brazil is dominated by the Cerrado biome, which spans over 2 million km² and is composed of a mosaic of phytophysiognomies, harboring high species diversity. It is considered one of the world’s biodiversity hotspots [44, 45]. This biome is extremely important for water resources, encompassing the headwaters and most of the river basins in South America, as well as the upper basins of the Amazon, Xingu, and Tapajós rivers [46].

Understanding the diversity and functions of sponge-associated microorganisms is essential for the conservation of these organisms and for the development of new biotechnological applications. Therefore, expanding studies on freshwater sponges, focusing on their symbiotic microorganisms, is crucial for understanding the functional roles of hosts and their symbionts in the environment, as well as for elucidating the evolutionary history between these groups. Furthermore, given the importance of the Cerrado biome for water resources, this work also aims to contribute to the understanding of the diversity of freshwater aquatic microorganisms and the importance of their preservation. In this work, we used a metagenomic approach to explore the microbiome of the sponge *Metania* sp., focusing on its diversity and functional potential.

## Methods

### Study area and environmental sample

**Fig. 1:**
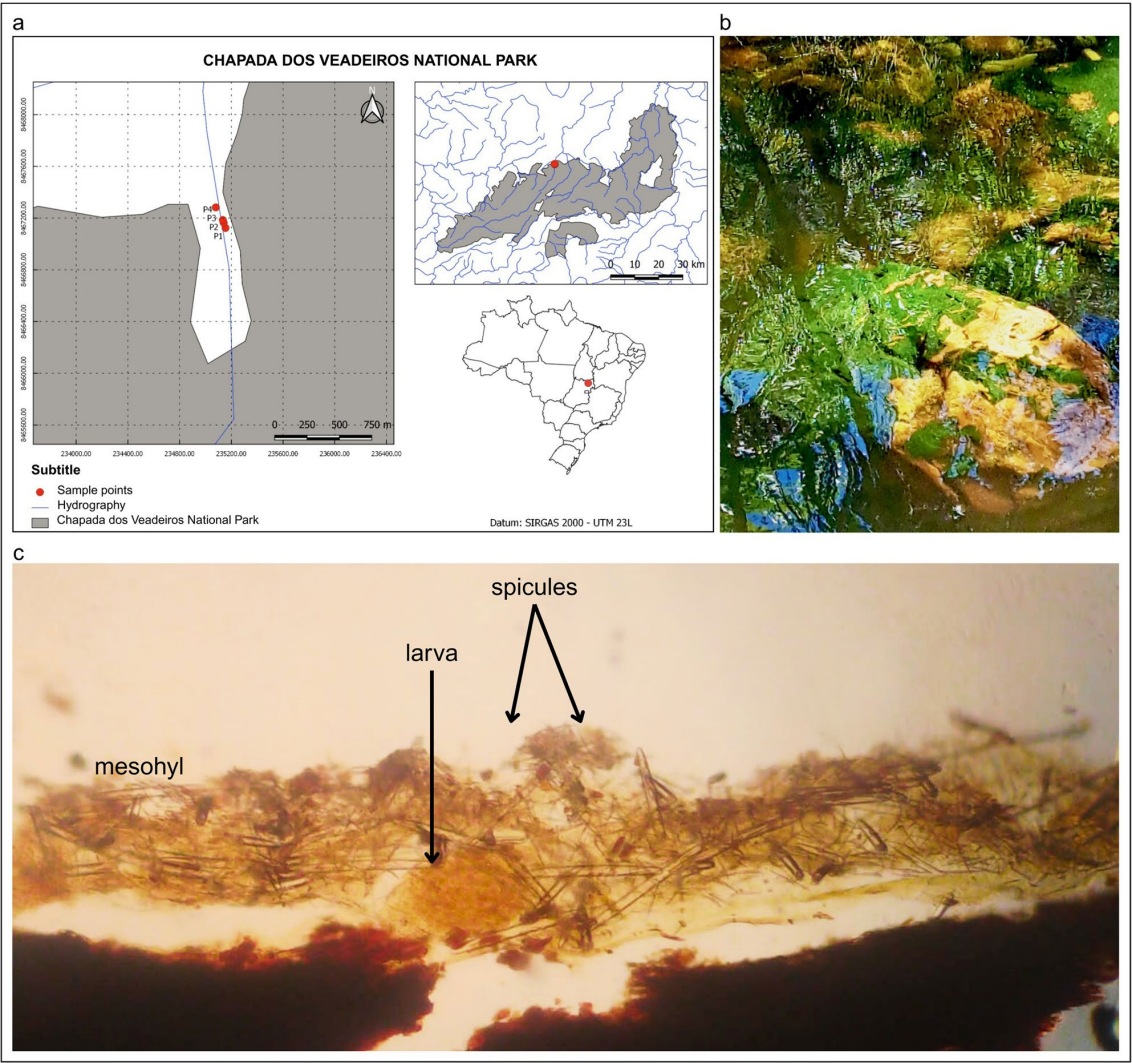
Data and images of the study area, located in Chapada dos Veadeiros National Park. **a** Map of the study area, indicating the location of Chapada dos Veadeiros National Park in Brazil (bottom map) and in the state of Goiás (top map). The main map details the collection area, showing the sampling points (P1-P4) and hydrography. **b** General view of the collection area, showing the sponge *Metania* sp. (green color) embedded in the rocks submerged in the river. **c** Optical microscopy image (100x magnification) of the sponge in reproductive phase, highlighting structures (larva, spicules, and mesohyl) with the arrow

The sampling was carried out, in the Veredas waterfall complex located in the Chapada dos Veadeiros, in the northern mesoregion of the state of Goiás (Cavalcante), Brazil in August 2023 (Fig. 1; Table 1). This complex is formed by diverse water bodies and is in the area adjacent to the Chapada dos Veadeiros National Park. It is in the Cerrado biome, presenting several phytophysiognomies, such as Gallery Forest, Dry Forest, Cerradão, Cerrado *sensu stricto*, Cerrado Park, Vereda, Dirty Field, Clean Field, and Rocky Field. The climate regime is semi-humid tropical of the Aw type, according to the Köppen classification, with two well-defined seasons: humid summer and dry winter [47, 48].

**Table 1:** UTM coordinates of sampling points and elevation (m), Veredas complex, Chapada dos Veadeiros National Park, Brazil

Points	Coordinates UTM (23 L)		Elevation
	X	Y	
P01	235155.3153	8467123.405	951
P02	235141.3864	8467163.334	756
P03	235134.4545	8467185.402	930
P04	235080.1374	8467282.479	937

The sponge samples were collected at four sampling points, by scraping the rocks where they were attached and placed in Falcon tubes and fixed in ethanol at 80%. Surrounding water samples for molecular analysis were collected in plastic bottles (1.5 L) by submerging the bottles in the subsurface. The sponge and water samples were transported to the temporary work basis, and the sponges were immediately frozen in dry ice. Water samples (500 mL) were filtered through 0.22 μm pore size Millipore nitrocellulose membranes and frozen in dry ice. All samples were done in triplicate.

Water temperature was measured in situ using an analogic thermometer (Incoterm), while pH, electrical conductivity, and dissolved solids were measured using field probes (Thermo Scientific Orion Star 3 portable pH meter, Hach SensION 5 portable Conductivity Meter). Water samples were collected in plastic bottles (properly washed with 50% HCl, tap water and distilled water) for analysis of nitrate, ammonia, phosphorus, total iron, and total suspended solids. Samples for analysis of nitrogenous forms and phosphorus were preserved with 1 mL of sulfuric acid (H_2_SO_4_) and 2 mL of nitric acid (HNO_3_), respectively. The samples were refrigerated at 4 °C until analysis in the laboratory.

### Physical and chemical parameters

#### Nitrogenous forms

Nitrate was determined by the Cadmium Reduction Method – low range, using HACH reagents (NitraVer6 and NitriVer3). For ammonia, the Nessler method was used, with a detection range of 0–2.5 mg/L NH_3_. Nitrate and ammonia concentrations were obtained by the colorimetric method, with a HACH DR2010 spectrophotometer, at a wavelength of 507 nm (nitrate) and 425 nm (ammonia).

#### Phosphorus

Phosphorus was measured in its reactive form, orthophosphate (PO4-3), by the PhosVer 3 method (Method 8048, Hach Company) with the Test’N Tube procedure. The procedure is equivalent to USEPA Method 365.2 and Standard Methods 4500-P for orthophosphate determination. In this method, orthophosphate reacts with molybdate under acidic conditions forming a phosphomolybdate complex, which is reduced by ascorbic acid to produce a blue-colored compound. The color intensity is proportional to the orthophosphate concentration and was measured by the colorimetric method, with a HACH DR2010 model spectrophotometer, at a wavelength of 890 nm.

#### Iron

The water sample was subjected to acid digestion with the addition of 2 mL of concentrated HCl and 1 mL of H_3_NO, heated until the volume was reduced. Subsequently, the pH was adjusted with 6 N NaOH to a range of 3–5. Method 8008 (HACH, USEPA Iron Ver) was used to measure iron, with reading on a HACH DR2010 spectrophotometer at 510 nm.

#### Total suspended solids

Total suspended solids were measured according to Standard Methods (2540 D. Total Suspended Solids Dried at 103–105 °C) [49]. In summary, the method consists of filtering a known volume of sample through a previously weighed glass fiber membrane, and the retained residue is dried for 24 h at 103–105 °C. The difference in weight of the membrane represents the total suspended solids.

### Genomic DNA extraction

The extraction of genomic DNA from the sponge was done in triplicate, using the commercial FastDNA Spin Kit for Soil (MP Biomedicals), according to the manufacturer’s protocol. DNA from the membranes (surrounding water) was extracted using the commercial DNeasy Power Water kit (QIAGEN), according to the manufacturer’s protocol, using a water bath at 65 °C for 10 min, in the cell lysis step. After extraction, 5 µL of DNA was applied to a 1.2% agarose gel to check the integrity of the fragments and quantified by QUBIT fluorometer Q32857 (Invitrogen). Genomic DNA was sent to Macrogen (Korea) for shotgun metagenomic sequencing.

### Library preparation and DNA sequencing

Libraries were constructed for each sample using the TruSeq Nano DNA kit (Illumina), with random DNA fragmentation, followed by ligation of 5’ and 3’ adapters at the ends. The libraries were sequenced using the Illumina NovaSeq6000 system (150 bp paired-end reads), yielding 21.9–28.0 Gbp of sequence data per sample (mean 24.1 Gbp).

### Metagenome assembly and taxonomic assignment

The reads were processed to remove adapters, contaminants and low-quality sequences using BBtools v 39.03 (https://sourceforge.net/projects/bbmap/). The assembly of the metagenomes was done separately, using MEGAHIT v 1.2.9 [50], with default parameters. The taxonomic assignment of the contigs was determined using SingleM (v 0.16.0) [51]. In this work, only the taxonomic profile of the microbiome and its respective abundances (coverage) were considered.

### Diversity analysis

The relative abundances of taxonomic groups were shown in bar plots. The Venn diagram identifies the number of shared groups among the sponge and surrounding water. All graphs were constructed using the *ggplot2* package [52] and *microbiome* (v 1.31.2) [53] in R software (v 4.3.3) [54, 55] .

To analyze richness and diversity, Shannon (diversity) and Pielou (evenness) index for each water and sponge microbiome were analyzed. Differences in diversity and evenness between the water and sponge microbiomes were evaluated by the nonparametric Kolmogrov-Smirnov test at a 95% significance level using the *microbiome* (v 1.31.2) and *vegan* (v 2.7.1) [56] packages in the R software. For beta diversity analyses, abundances were transformed (logx + 1) to normalize the data distribution. Beta diversity analysis was performed using Bray-Curtis distance matrices and Principal Coordinates Analysis (PCoA) ordination method, visualized in a two-dimensional ordination plot, using the *phyloseq* package (*ordinate* and *plot_ordination* functions) [57] in the R software. To assess statistically significant differences between microbiome groups, a Permutational Multivariate Analysis of Variance (PERMANOVA) was conducted using the adonis2 (v 2.7.2) function from the *vegan* package with 999 permutations and a significance threshold of α = 0.05. The top contributors to Bray-Curtis dissimilarities for taxa were identified using the *coef* function from the *stats* package (v 4.5.0) and visualized as a bar plot.

### Functional annotation

The open reading frames (ORFs) were predicted with Prodigal (v 2.6.3) [58] in –meta mode. The contigs were mapped using Bowtie2 (v 2.5.4) [59], indexed with Samtools (v 1.19.2) [60] and the genes coverage were calculated using CoverM (v 0.7.0) [61]. The metagenome assemblies were annotated using eggNOG-mapper (v 2.1.12) [62] with eggNOG database (v 5.0.2) [63] and KEGG (Kyoto Encyclopedia of Genes and Genomes) annotations were derived from the eggNOG-mapper results. For subsequent analyses, the annotations from eukaryote and virus sequences were filtered out. To identify differentially abundant KEGG Orthologs (KOs) between *Metania*-associated and water microbiomes, we performed a differential abundance analysis using DESeq2 package (v 1.49.4) [64]. All the KOs were used to identify the enrichment of KEGG metabolic pathways in both microbiomes using the *enrichKegg* function of the *clusterProfiler* package (v 4.16.0) [65–68] in R software. We also search for BGCs by antiSMASH tool (v 7.0) [69] and compared them with the Minimum Information about a Biosynthetic Gene cluster (MIBIG) repository, which stores gene clusters of natural products with known functions and experimentally characterized. Biosynthetic gene clusters (BGCs) predicted by antiSMASH were clustered into gene cluster families (GCFs) using BiG-SCAPE v2.0 [70] with a distance cutoff of 0.3, using the mix mode and including singleton clusters. Similarity networks were anchored to the MIBiG database (v 3.1) [71].

## Results

### Characterization of physical and chemical parameters in the aquatic environment

To characterize the aquatic environment in which the freshwater sponge *Metania* sp. was sampled, we measured the environmental variables (Table 2). The water temperature was 21.5 ± 0.58 °C, pH was in the neutral range (6.6 ± 0.12), and electrical conductivity varying around 5.16 ± 0.07 µS/cm². Total dissolved solids (TDS) presented the same concentration at all points, 2.2 mg/L. However, total suspended solids (TSS) presented an average value of 5.85 ± 6.07 mg/L, with the highest concentration at point P03. The concentration of ammoniacal nitrogen (NH_3_ – N) was 0.04 ± 0.02 mg/L, with higher values at sampling points P03 and P04 (0.06 mg/L). Nitrate (NO_2_ – N) had the same concentration at all sampling points (0.01 mg/L). Phosphorus, in the form of orthophosphate (PO_4_^3−^), had an average of 0.11 ± 0.03 mg/L, with the higher concentration at point P04, while iron (Fe) was higher at point P03 (1.33 mg/L), with an average of 0.85 ± 0.34 mg/L.

**Table 2:** Environmental variables (mean and standard deviation) of the sampling points (Point 01–04), Brazil (2023)

Variables	P01	P02	P03	P04	Mean	Standard-deviation
Water temperature (°C)	22.00	22.00	21.00	21.00	21.50	0.58
pH	6.50	6.50	6.70	6.70	6.60	0.12
Electric conductivity (µS/cm²)	5.22	5.07	5.21	5.13	5.16	0.07
Total dissolved solids (mg/L)	2.20	2.20	2.20	2.20	2.20	0.00
Total suspended solids (mg/L)	0.40	5.90	14.30	2.80	5.85	6.07
Ammonia nitrogen (mg/L)	0.04	0.01	0.06	0.06	0.04	0.02
Nitrate (mg/L)	0.01	0.00	0.01	0.01	0.01	0.00
Orthophosphate (mg/L)	0.08	0.11	0.12	0.14	0.11	0.03
Iron (mg/L)	0.71	0.81	1.33	0.54	0.85	0.34

### Metania sp.-associated microbiomes contain groups common to other sponges

We performed metagenomic sequencing of the freshwater sponge *Metania sp*. and the surrounding water to compare the microbial communities. Approximately 1.2 billion reads were retrieved from the eight sequenced metagenomes, with Phread quality of 95% (Q20) and average GC content of 46.3% and 51%, for *Metania* sp. and surrounding water metagenomes, respectively. The reads from each metagenome were assembled separately into contigs, resulting in 4 million contigs, with average N50 of 1,627 bp (Table 3).

**Table 3:** Summary of sequencing and assembly statistics for the *Metania* sp. and surrounding water metagenomes

Samples	*N*º bases (Mbp)	reads *N*º (Mi)	GC (%)	Q20 (%)	Contigs *N*º	Contig Length (Mbp)	N50 (bp)
S-01	21,982	146	45.4	94.7	463,535	370	1804
S-02	23,363	155	45.5	94.5	473,243	370	1720
S-03	28,014	186	47.3	93	488,921	389	1856
S-04	24,196	160	47.2	93.5	435,645	345	1780
W-01	25,651	170	51.1	94.5	503,051	392	1444
W-02	22,282	148	50.9	95.1	450,404	372	1491
W-03	23,751	157	50.7	95.2	390,127	341	1988
W-04	23,784	158	51.3	95.5	892,047	645	936
Total	193,022	1.278			4,096,973		
Mean	24,128	160	49	95	512,122	403	1627

Columns include the number of bases sequenced (Mbp), number of reads (Mi), GC percentage, sequencing quality (Q20), number of contigs, average contig length (Mbp), and N50 value (bp). Table rows represent individual samples, followed by totals and averages for all samples

**Fig. 2:**
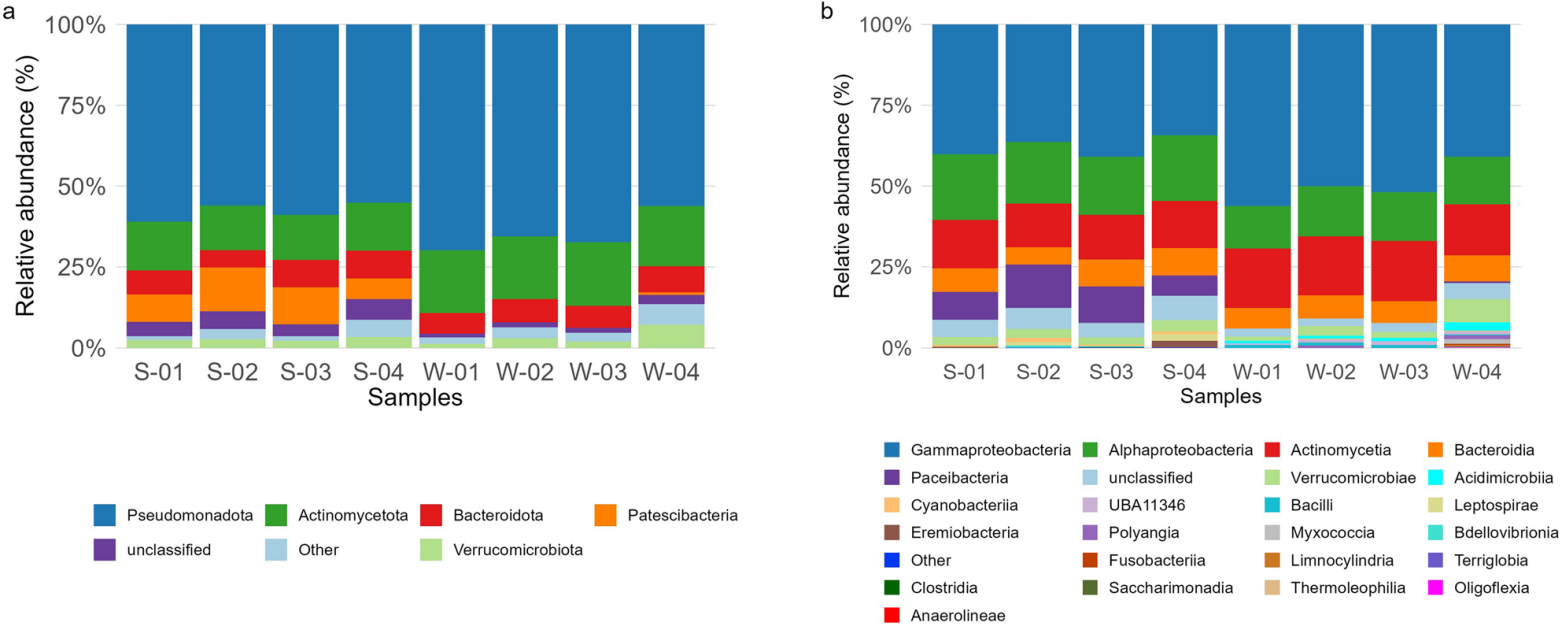
Taxonomic composition of microbiome associated with *Metania* sp. (S-01 to 04) and the surrounding water (W-01 to 04). The samples were collected at the Veredas waterfall complex, Chapada dos Veadeiros, Goiás State, Brazil. **a** Relative abundance (%) of phyla; the phyla are represented by different colors, as indicated in the legend at the bottom of the figure. **b** Relative abundance (%) of different classes; the classes are represented by different colors, as indicated in the legend at the bottom of the figure

The assembled contigs were classified into 243 taxonomic units, forming distinct hierarchical ranks across all samples. We identified 17 phyla, 23 classes, 45 orders, 69 families, 106 genera, of which 19 were further classified to the species level. Considering all the taxonomic units classified, 241 were bacteria with approximately 99% of the relative abundance, and two were Archaea (~ 1%). Archaea were represented by *Thermoproteota* and an unclassified phylum. In the *Metania* sp. microbiome, the most abundant phyla were *Pseudomonadota* (57%), *Actinomycetota* (14%), *Patescibacteria* (CPR group, 9.9%) and *Bacteroidota* (7.5%), while *Verrucomicrobiota* (2.7%) and *Cyanobacteriota* (0.8%) were present in lower abundance (Fig. 2a). The free-living microbiome from the surrounding water was represented by *Pseudomonadota* (64.8%), *Actinomycetota* (19.2%), and *Bacteroidota* (7.1%). *Planctomycetota* (0.9%) and *Bacillota* (0.8%) occurred in the surrounding water but were absent in the sponge’s microbiome. The dominant classes were common in both microbiomes (*Metania*-associated and water), with *Gammaproteobacteria* (44%), and *Alphaproteobacteria* (17%) (within *Pseudomonadota*), *Actinomycetia* (16%, within *Actinomycetota*); *Acidimicrobiia* occurred only in the water microbiome (1.3%) (Fig. 2b). *Verrucomicrobiae* and *Cyanobacteriia* were relatively low in abundance and frequency. 

### The microbial community structure associated with *Metania* sp. is different from the surrounding water

**Fig. 3:**
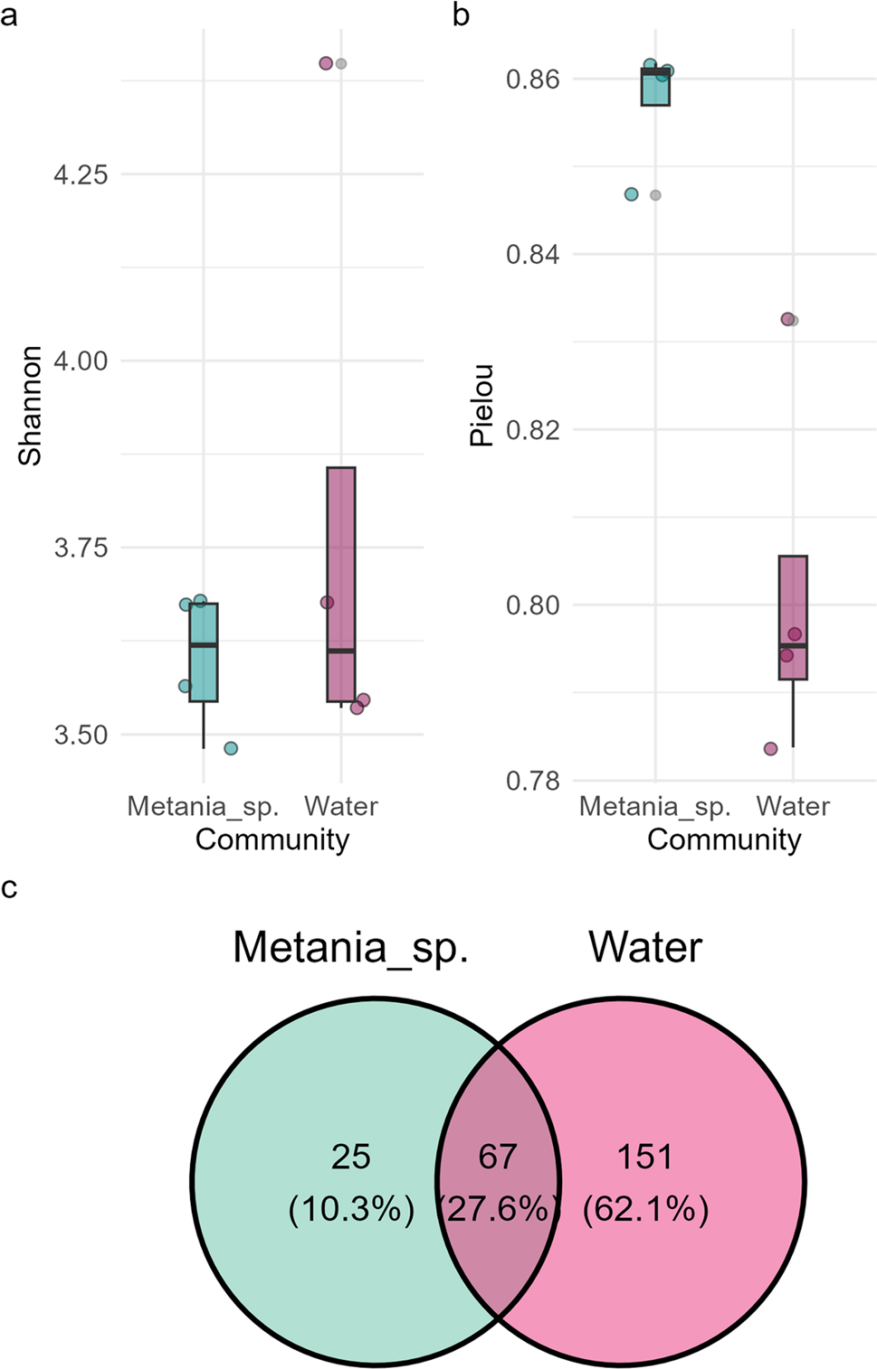
Alpha diversity metrics between microbiomes originating from the sponge (green) and the surrounding water (rose).** a** Shannon index represents the diversity of the communities (Kolmogrov-Smirnov test; D = 0.5; *p* = not significant). **b** Pielou index indicates the evenness of species within the communities, with values closer to 1.0 indicating greater evenness (Kolmogrov-Smirnov test, D = 1.0, *p* < 0.05). **c** Venn diagram of taxa microbiome unique or shared between sponge and surrounding water

**Fig. 4:**
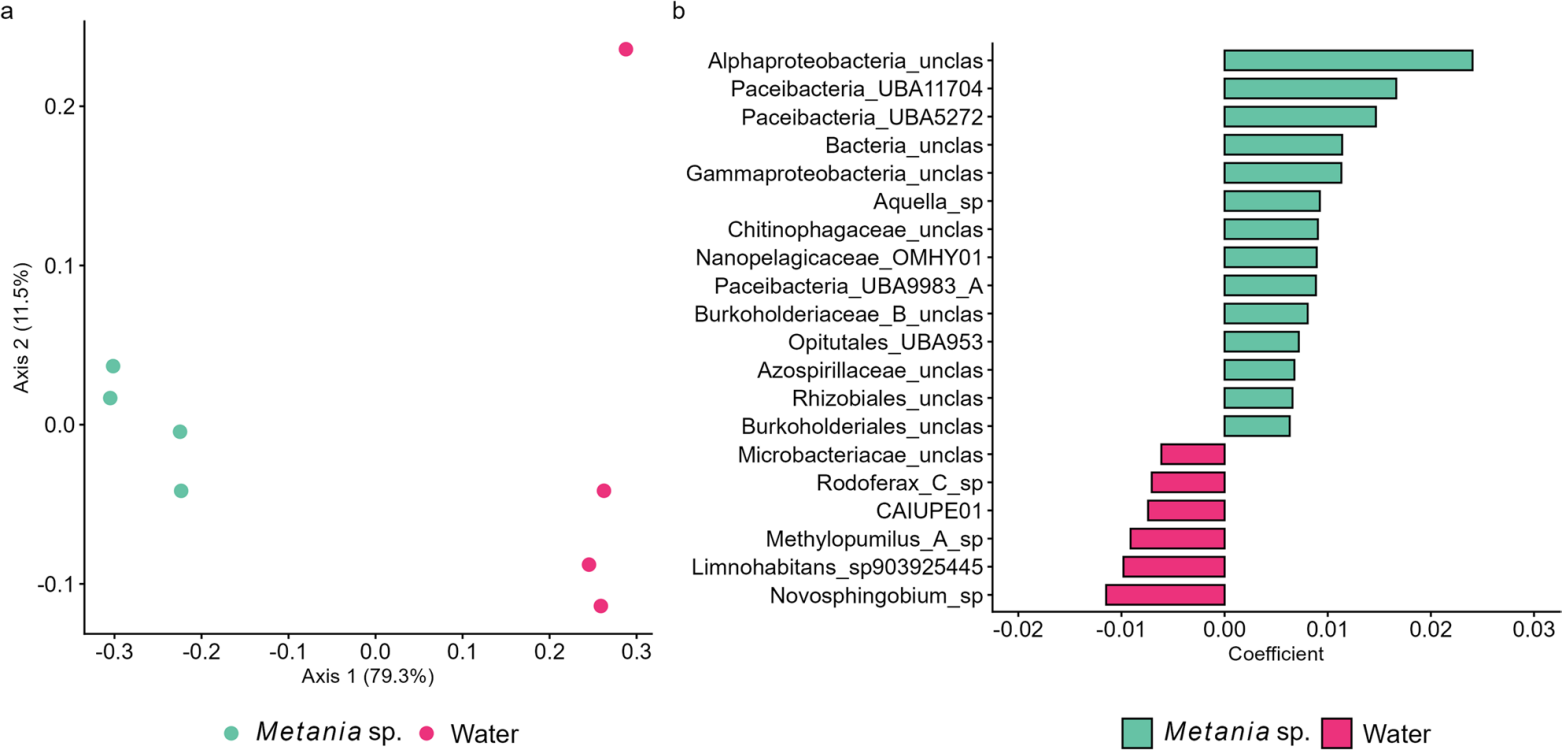
Beta diversity of the microbiomes associated with *Metania *sp. (green) and the surrounding water (rose). **a** Principal Coordinates Analysis (PCoA) using the Bray-Curtis dissimilarity index (*R*² = 0.71, *p* = 0.04) (PERMANOVA; F = 21.6, *df* = 1, *R*² = 0.78, *p* = 0.029). Each point represents one sample. **b** Representation of the 20 largest species contribution coefficients to beta diversity, extracted from PERMANOVA. The positive and negative values are the coefficients of microbiome groups associated with *Metania* sp. and with the surrounding water, respectively. Unclas = unclassified taxa

To compare the microbial community structure of *Metania* sp. and water, we calculated Shannon diversity, evenness, and beta diversity indices. The Shannon alpha diversity demonstrated no significant difference (Kolmogrov-Smirnov test; D = 0.5, *p* = 0.771) among the *Metania*-associated (3.59 ± 0.09) and water microbiome (3.78 ± 0.38) (Fig. 3a). Pielou’s evenness index, which indicates the presence of dominant taxa in the communities, was higher in *Metania* sp. (0.85 ± 0.007) than in water (0.80 ± 0.032) (Kolmogrov-Smirnov test, D = 1.0, *p* = 0.028) (Fig. 3b). Considering the 243 classified taxa, 25 were exclusive to *Metania*-associated microbiome (10.4% of relative abundance), 151 to water (32.4% of relative abundance) and 67 (89.6% and 67.6% of relative abundance in sponge- and water-associated microbiome, respectively) were shared among both (Fig. 3c). The beta diversity indicated by Bray-Curtis dissimilarity showed that the composition of the *Metania*-associated and water microbiome is clearly distinct (PERMANOVA; F = 21.6, *df* = 1, *R*² = 0.78, *p* < 0.04) (Fig. 4a). The 20 coefficients extracted from PERMANOVA indicated the contribution of the taxa to beta diversity, showing groups of *Alphaproteobacteria* and *Patescibacteria* contribute greater to the differentiation of the microbiomes (Fig. 4b). *Patescibacteria* was detected in all sponge metagenomes but occurred just in one water sample in low relative abundance (< 1%).

### Differential functional profiles between sponge- and water-associated microbiomes

**Fig. 5:**
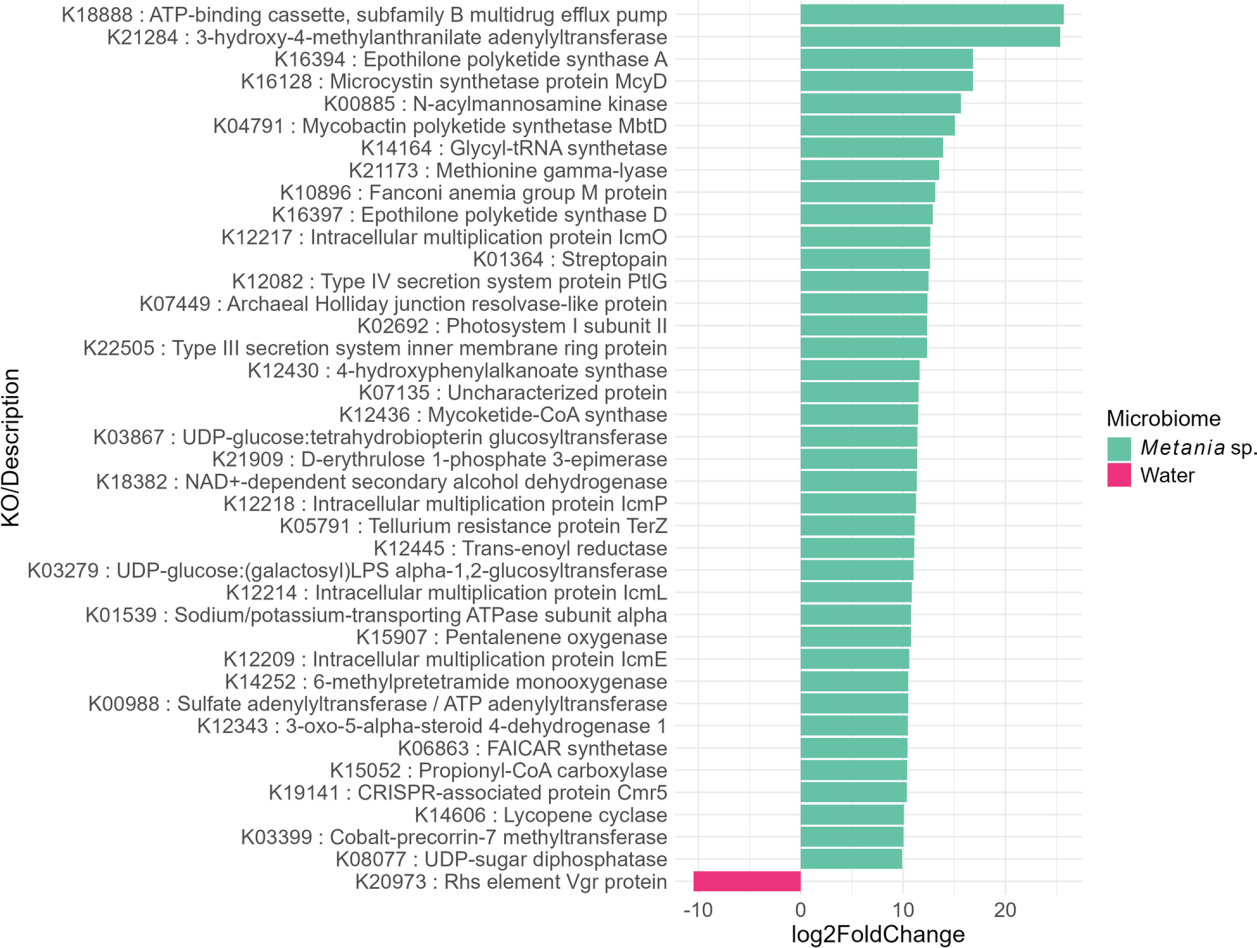
Differential abundance analysis of KEGG Orthologs between the sponge and the water microbiome. The x-axis represents the log2-fold change, where positive values (green bars) indicate KOs enriched in the *Metania* sp. microbiome and negative values (rose bars) indicate KOs enriched in the water samples. The y-axis lists the specific KEGG Orthologs (KO) IDs and descriptions

We functionally annotated the genes predicted by Prodigal with eggNOG, resulting in 7,201 KOs from prokaryotes. Differential abundance analysis revealed KOs that were significantly more abundant in one group compared to another. We identified 2,299 (41%) differentially abundant (DA) KO (*p-adj* < 0.05 and |log2FC| > 1), with 1,275 overrepresented KOs in the water microbiome and 1,024 depleted in this group. The absolute values of log2FC ranged from 1 to 25, and among the 40 top values, 39 correspond to genes more abundant in the *Metania*-associated microbiome (Fig. 5).

The KOs enriched in sponge microbiomes were related to secretion systems III (K22505) and IV (K12209, K12214, K12217, K12218, K12082), which are involved in exporting proteins to the outer membrane [72]. Furthermore, KOs associated with genetic processing, such as the Holliday junction resolvase-related endonuclease (K07449) and the C-terminal domain of the helicase superfamily (K10896), which were previously found in archaea [73]. Glycyl-tRNA synthetase (K14164), which ensures the correct amino acid is incorporated into the protein, was enriched in the sponge microbiome. Other abundant KOs found in the sponge microbiome are involved in functions such as metabolism of cofactors and vitamins (K03399, cobalt-precorrin-7 (C5)-methyltransferase), carotenoids (K14606, lycopene cyclase), and siderophores (K04791, mycobactin polyketide synthetase). Genes that participate in defense system pathways are also abundant, among them CRISPR-associated proteins (K19141, *cmr5*). Furthermore, we recorded abundant genes that encode secondary metabolites, such as those involved in the biosynthesis of several antibiotics, including sibiromycin (K21284, 3-hydroxy-4-methylanthranilate adenylyltransferase), calicheamicins (K21173, methionine gamma-lyase), pentalenolactone (K15907, pentalenene oxygenase), tetracycline (K14252, 6-methylpretetramide 4-monooxygenase / 4-hydroxy-6-methylpretetramide 12a-monooxygenase). We also identified KOs that encode a polypeptide from the subunit II of photosystem I (*psaD*, K02692), and a microcystin synthetase protein (*mcyD*, K16128).

**Fig. 6:**
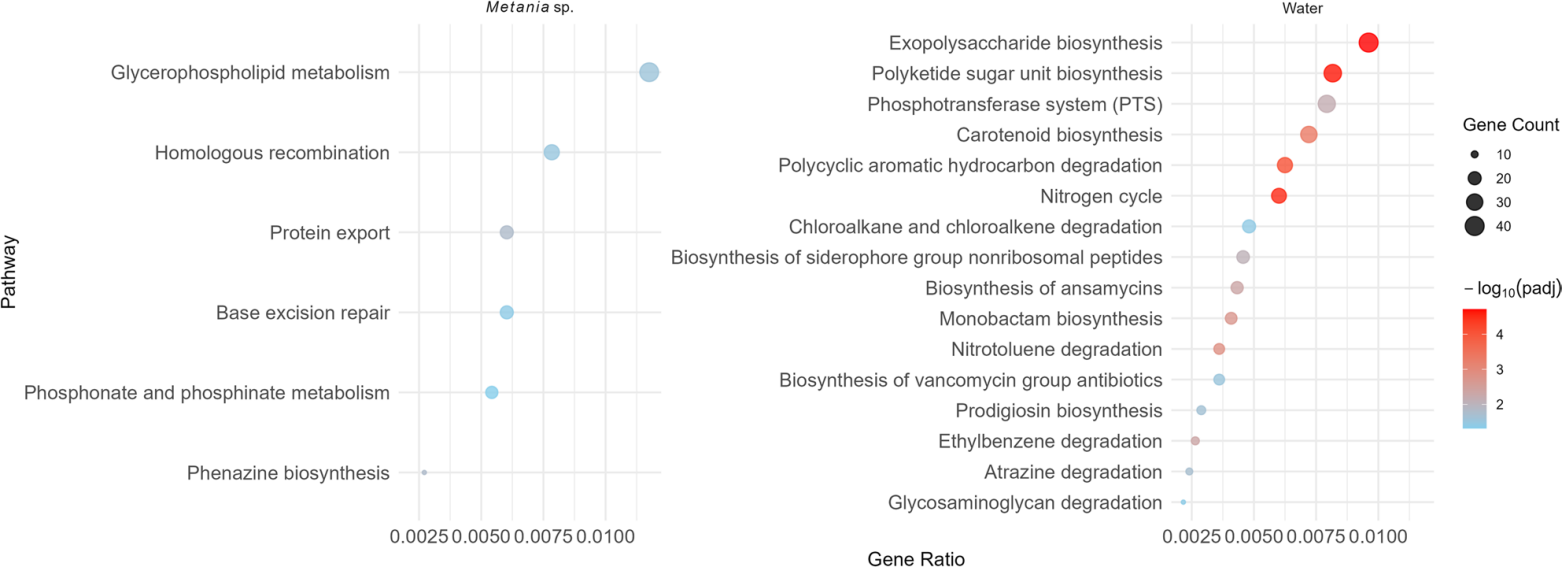
Enrichment of unique KEGG metabolic pathways in *Metania* sp- and surrounding water-associated microbiomes. Bubble size represents the count of orthologous genes. Colors represent log p-adj (*enrichKEGG*), where red is most significant and blue is least significant

While the differential abundance analysis identifies individual KOs that vary between microbiomes, pathway enrichment analysis provides a higher-level functional perspective by assessing whether these genes are associated with broader metabolic pathways. We identified 101 and 111 significantly enriched KEGG pathways (*p-adj* < 0.05) in the *Metania* sp. microbiome and the surrounding water, respectively (Fig. S1). The 95 pathways identified as simultaneously enriched in both microbiomes are part of the core microbial metabolic functions, such as the biosynthesis of amino acids, carbon metabolism, the biosynthesis of nucleotide sugar, and the biosynthesis of cofactors. Six pathways were significantly enriched in the *Metania*-associated microbiome only (Fig. 6). Among these, three pathways associated with genetic information processing including base excision repair (BER), homologous recombination (HR), and protein export, which reinforces the record of DA KOs in *Metania* sp. Furthermore, pathways for glycerophospholipid, phosphonate and phosphinate, and phenazine biosynthesis were also enriched in *Metania* sp. microbiome.

In contrast, 16 metabolic pathways were significantly enriched (*p-adj* < 0.05) just in the water-associated microbiome (Fig. 6). These are pathways of exopolysaccharide biosynthesis, terpenoids, polyketides, and secondary metabolites (polyketide sugar unit, carotenoids, prodigiosin, ansamycins, vancomycin group antibiotics, and monobactam). In addition to these, functions related to nutrient cycling, such as nitrogen cycle and the phosphotransferase system were also enriched. Other significantly enriched pathways included the degradation pathways of complex organic pollutants and xenobiotics, such as polycyclic aromatic hydrocarbon, nitrotoluene, chloroalkane and chloroalkene, and atrazine.

### Microbiomes are potential producers of natural products

**Fig. 7:**
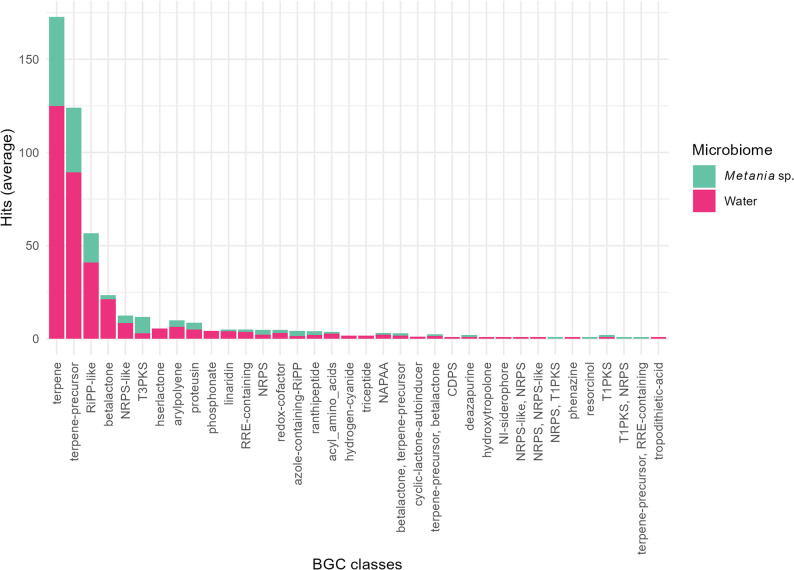
Number of hits from biosynthetic gene clusters (BGCs) from *Metania* sp. and water microbiome. The x-axis shows different types of BGCs, and the y-axis shows the average number of hits within each microbiome for each BGC type

To identify the types of natural products that are potentially produced by *Metania* sp. and the water microbiomes, we mined the datasets using antiSMASH. Across all the metagenomes, 1,891 BGCs were predicted, spanning 36 distinct biosynthetic classes, with 32 classes detected in water and 24 in sponge-associated microbiomes. The most frequent classes in water were terpene (500 BGCs), terpene-precursor (357 BGCs), ribosomally synthesized and post-translationally modified peptides (RiPP-like, 164 BGCs) and betalactone (85 BGCs). Type III Polyketide synthases (T3PKS) were most abundant in *Metania*-associated microbiome (35 BGCs) (Fig. 7). To assess biosynthetic diversity beyond broad classifications, BiG-SCAPE clustering was performed, resolving the 1,891 individual BGCs into 542 distinct Gene Cluster Families (GCFs). Given the fragmented nature of the assemblies, with many clusters potentially located at contig edges, this elevated number of GCFs should be interpreted cautiously, as it may reflect partial clusters rather than true biosynthetic diversification. 118 GCFs were exclusive to the sponge-associated microbiome, 315 were exclusive to the surrounding water, and 109 were shared between both microbiomes (Supplementary Table 1). Exclusive GCFs from both environments were predominantly associated with terpenes (72 in *Metania* sp. and 156 in water), indicating that terpene-associated families are frequently detected in the metagenomes analyzed.

For analysis of the similarity of predicted BGCs with the MIBIG repository, hits of low (30–75%) and high similarity (> 75%) were considered, totaling 92 hits (19 in *Metania* sp. and 73 in the water microbiome) (Tab. S1). These BGCs show similarity to 10 known compounds, such as pigments (carotenoid, zeaxanthin), polyester (polyhydroxyalkanoate), antimicrobial (paenibacterin, icosalide A/B, macolacin), and antifungal (mycosubtilin, vioprolide A/B/C). Two metabolites that are not typically associated with bacteria were matched, retigeranin/arathanatriene, a plant-derived sesterpene, and clavaric acid, fungi-derived with antitumor properties. The 40 high-similarity hits code for five distinct named compounds, most of them are carotenoids (9 hits/sponge, 26 hits/water), in addition to zeaxanthin (2 hits/water), clavaric acid (1 hit/water), icosalide A/B (1 hit/water), rhizomide A/ B/C (1 hit/water).

These results agree with the enriched KOs in each microbiome. For example, in the surrounding water, we recorded differentially abundant KOs involved in carotenoid (K06443; *lcyB*/ K20616; *lyeJ*) and terpenoid (K06013/ K21275; *hexs-b*/ K00054; *mvaA*/ K01597; MVD, *mvaD*) biosynthesis pathways. In the sponge microbiome, enriched KOs related to BGC were found, including epothilone PKS synthase A (K16394; *epoA* and K16397; *epoD*) and mycobactin PKS synthase (K04791; *mbtD*).

## Discussion

### Taxonomic composition and diversity

This work characterized the taxonomic and putative functional profile of the microbiota associated with the freshwater sponge *Metania* sp. using metagenomic approaches. The functional roles discussed below are inferred from previously reported metabolic traits of these taxa in the literature and are used here to provide ecological context for the taxonomic composition observed in the *Metania* sp. microbiome.

The phylum *Pseudomonadota* dominated the microbial community of *Metania* sp., corroborating previous investigations of both marine and freshwater sponges [22, 23, 25, 43, 74]. Beta diversity analysis revealed distinct differences between the *Metania* sp.-associated microbiome and the microbiome of the surrounding water. Despite sharing only 27% of taxa, these groups represented more than 80% of the total microbial abundance. This pattern is consistent with previous insights on the acquisition of microorganisms by horizontal transmission from the water column to sponge [75, 76] coupled with the influence of the host on the selection of microbial symbionts [22].

The composition of the microbiome of *Metania* sp. exhibited similarity to other freshwater and marine sponges, suggesting conserved patterns of microbial association. Specifically, the dominance of phylum *Pseudomonadota* aligned with microbiomes of other freshwater sponges such as *Spongilla lacustris*, *Ephydatia fluvialis*, *Corvospongilla lapidosa*, *Tubella variabilis*, *Heteromeyenia cristalina*, and *Metania reticulata* gemmules [14, 22, 23, 25, 33, 43] as well as that of marine sponges [74]. This consistent prevalence of *Pseudomonadota* across diverse sponge hosts underscores its significant ecological and metabolic roles within the holobiont. Members of this phylum are widely reported in the literature to display metabolic versatility, participating in a wide array of biogeochemical cycles and possessing catabolic capabilities [77, 78], which may contribute to nutrient acquisition and environmental adaptation in sponge-associated systems.

Within *Pseudomonadota*, the class *Gammaproteobacteria* was predominantly represented by the order *Burkholderiales*. Members of this order have been previously described as possessing catabolic potential in the degradation of aromatic compounds [79]. Based on these reported capabilities, their presence in the *Metania* sp. microbiome may indicate a potential bacterial role in the detoxification of the sponge’s internal environment from potentially harmful aromatic pollutants or in accessing carbon and energy from recalcitrant organic matter filtered from the surrounding water [14]. Among the *Alphaproteobacteria*, the order *Sphingomonadales* include organisms reported to degrade mono- and polycyclic aromatic compounds, and that have been explored for bioremediation applications [80]. Such metabolic capabilities described in previous studies may contribute directly to the resilience and nutritional strategies of the sponge holobiont.

The phyla *Actinomycetota* and *Bacteroidota* were also well represented in the sponge microbiome, consistent with other sponge-associated microbiome investigations [14, 20, 43, 81]. These bacterial groups have been widely reported to participate in the synthesis of bioactive compounds, degradation of aromatic components [66] and in biogeochemical processes, particularly through their functional roles in the decomposition of peptides and polysaccharides [82].

The presence of the superphylum *Patescibacteria* represents another interesting finding in the *Metania* sp. microbiome. This group is characterized by reduced genomes and limited biosynthetic capabilities [83], traits often associated with a symbiotic lifestyle, including potential parasitism and methanogenic interactions, as observed in the class *Paceibacteria* [84]. The detection of *Patescibacteria* in *Metania* sp. corroborates previous records in both marine (*Vazella pourtalesii*) [85] and freshwater (*Spongilla lacustris*) sponge species, where their presence has been associated with organic chlorine contaminants such as dieldrin [32].

Despite its lower representation in our study, the phylum *Verrucomicrobiota* is a diverse and common component in sponge microbiomes [24, 31, 36, 37, 43], with the order *Opitutales* frequently occurring in these associations [36]. Members of this group have been described as possessing both aerobic and anaerobic metabolic capabilities and are known for their potential to degrade polysaccharides [86] and perform acidophilic methanotrophic activity [87].

The phylum *Cyanobacteria*, frequently reported as one of the most abundant in the sponge-microorganism association [18, 19, 25], exhibited low relative abundance in our study. This pattern may reflect their low abundance in the surrounding water column. Cyanobacteria are well known as primary producers in aquatic environments and as producers of toxins and bioactive secondary compounds [88, 89].

### Functional diversity

Functional prediction of the microbiomes associated with *Metania* sp. and surrounding water revealed an extensive metabolic diversity, with enriched metabolic pathways in the sponge microbiome associated with growth, nutritional support, defense, and adaptation of the holobiont. Both microbiomes exhibited important functions including metabolism of various amino acids (essential for the construction of proteins) and primary pathways of carbohydrate metabolism (important for energy and carbon acquisition through sugar processing). However, the sponge-associated microbiome appeared specialized in functions to survive in the host, including the genomic stability. The enriched pathways and KO groups in *Metania* sp. linked to genetic information processing, such as base excision repair and homologous recombination, suggest mechanisms important to maintain genomic integrity [90]. Sponges and their symbionts in shallow tropical environments experience oxidative stress due to variations in temperature, UV radiation, and oxygen levels, making these repair pathways essential [91, 92]. Interestingly, enrichment of these putative functions was also found in *Hymeniacidon* sp.-associated microbiome in shallow hydrothermal vents [93]. This is consistent with a convergent adaptative strategy among sponge-associated microbiomes under stress conditions, reinforcing the importance of these biological functions in maintaining microbial populations. Furthermore, these pathways facilitate adaptive processes, including acquisition of resistance mechanisms and increased genomic plasticity driven by recombinational repair, which fundamentally impacts prokaryotic core genome evolution and long-term adaptation [94–97].

#### Lipid metabolism and nutrient cycling

We observed significant enrichment of the glycerophospholipids metabolism pathway in the *Metania* sp. microbiome. This pathway involves the synthesis, remodeling, and degradation of key structural and signaling molecules [98], including phosphatidylcholine (PC), phosphatidylethanolamine (PE), phosphatidylserine (PS), phosphatidylinositol (PI), phosphatidylglycerol (PG). The enrichment of this pathway aligns with the observed taxonomic dominance of *Pseudomonadota*, with the prevalence of genes for PE and PG synthesis likely reflecting the typical membrane composition of this phylum [99]. Genin et al. [100] previously hypothesized that the high levels of PG and PI in marine sponge tissue are derived from their bacterial symbionts, as these molecules are characteristic of bacterial membranes (containing branched, short-chain, monounsaturated acids). Although several studies have characterized the lipidome of freshwater and marine sponges, noting their adaptive role in stress conditions and bioactive defense [100–104], the contribution of symbiotic bacteria to glycerophospholipid synthesis remains an active area of investigation.

The sponge microbiome plays an important role in nutrient cycling, particularly for nitrogen, phosphorus, and sulfur. Phosphonate and phosphinate metabolism, enriched in *Metania* sp. microbiome, enables the assimilation, degradation, and utilization of compounds containing carbon-phosphorus (C–P) bonds as alternative sources of phosphorus, especially in nutrient-limited environments [105, 106]. According to Yao et al. [107], *Alphaproteobacteria*, *Gammaproteobacteria*, and likely *Actinobacteria* can acquire phosphonate compounds and cleave the C-P bonds to obtain phosphorus, particularly in inorganic phosphate (Pi) limitation. However, research reviewed by Ruffolo et al. [105] using cultivation, metagenomics, and transcriptomics approaches has shown that phosphonates can be consumed as sources of nitrogen or carbon even at abundant phosphate levels.

Freshwater environments are typically limited in inorganic phosphorus availability, which is essential to primary production. However, we measured approximately 0.11 mg/L of orthophosphate in the surrounding water, which according to Class I water quality standards [108] is slightly above the classification limit (0.1 mg/L). Phosphorus is important not only for the symbiotic bacteria but also for the host sponge. Polyphosphates (poly-P) granule production and storage by symbiotic microbes have been identified in marine sponge, potentially serving as energy storage and protecting the holobiont during periods of phosphate scarcity [19, 109, 110].

#### Defense and adaptation systems

Our results revealed abundant KO groups related to microbial defense systems. These functions have been recorded in other sponge microbiomes and are considered indicative of symbiosis-related functions [26, 111]. The type III and IV secretion systems (T3SS and T4SS, respectively) translocate bacterial effector proteins into the host cell cytosol and are associated with bacterial pathogenesis in eukaryotic hosts [112, 113]. The T3SS may facilitate bacterial entry into host cells and modulate cell signaling processes and the immune response, evading phagocytosis and facilitating host colonization [114, 115]. T4SS has been documented in the microbiomes of marine sponges *Spongia officinalis* and *Ephydatia muelleri* [26, 111]. Virulence-associated plasmids containing structural modules with conjugative functions containing T4SS have been identified in *Vibrio crassostreae* [116].

Clustered regularly interspaced short palindromic repeats (CRISPR), a characteristic feature of most bacterial and archaeal genomes, provide resistance against phages when associated with *cas* genes [117]. Homologous recombination (HR), mediated by the RecA protein, is a central mechanism for spacer acquisition in the CRISPR-Cas system. During phage infection, homologous recombination integrates fragments of viral DNA into the CRISPR locus of the bacterial genome, conferring adaptive immunity [118–121]. These functional characteristics were enriched in the sponge microbiome, supporting the hypothesis that bacterial defense systems against foreign DNA and viruses are important for maintaining the host microbiome and highlighting an evolutionary characteristic of the symbiosis. Enrichment of defense systems has been found in Mediterranean marine sponge-associated microbiomes [122]. In the freshwater sponge *Ephydatia muelleri*, the family *Comamonadaceae* was enriched in CRISPR genes [26]. Karimi et al. [111] found that CRISPR-Cas proteins had higher frequencies in *Spongia officinalis* than in the marine environment. In a separate study, we examine the diversity and potential roles of viral communities associated with *Metania* sp. [123].

#### Biotechnological potential

We identified KOs involved in the synthesis of several compounds with antimicrobial activity, including sibiromycin, tetracycline, calicheamicin, and pentalenolactone. The phenazine biosynthesis pathway, detected in the *Metania* sp. microbiome, produces redox-active molecules that generate toxic reactive oxygen species through the direct reduction of molecular oxygen, accounting for their broad-spectrum antibiotic activity and role as virulence factors [124, 125]. This function has been documented in bacteria isolated from marine sponges, which showed the presence of the gene clusters for phenazine synthesis and gene expression associated with antimicrobial activity [126–128].

We identified BGCs in both the *Metania* sp. microbiome and the microbiome of the surrounding water, including non-ribosomal peptide synthetases, RiPPs, polyketides, terpenes, and betalactones, which encode pigments, antimicrobials, and antifungal agents. Although the water microbiome harbored a higher number of BGCs, the sponge microbiome also demonstrated biotechnological potential for producing secondary metabolites with diverse biological activities, including antimicrobial agents that may aid in host defense against predation and pathogens. Clark et al. [42] verified the production of several secondary metabolites by bacteria isolated from the freshwater sponge *Eunapius fragilis*. The microbiome of freshwater sponge *Spongilla lacustris* also exhibited high biosynthetic potential, verified through both metagenomic and cultivation approaches [31]. Secondary metabolite production may play roles in intra- and interspecific bacterial defense, cell signaling, and conferring host defense against pathogens and predation.

## Conclusions

In summary, this study demonstrates that the freshwater sponge *Metania* sp. hosts a complex and functionally specialized microbial community that plays fundamental roles in the adaptation, nutrition, and defense of the holobiont. The findings significantly advance our understanding of symbiotic relationships in freshwater sponges and provide insights into the evolutionary conservation of sponge-microbe associations across marine and freshwater environments. The identified metabolic capabilities, particularly those related to nutrient cycling, stress response, and bioactive compound production, highlight the ecological importance of these symbioses and their potential biotechnological applications. While this study establishes a comprehensive ecological overview of the microbiome, specific taxon-function attributions will be addressed in a subsequent analysis based on metagenome-assembled genomes. We consider this genomic reconstruction essential to ensure robust functional linking, thereby avoiding the potential inaccuracies inherent in analyses relying solely on short contigs or unassembled reads.

## Supplementary Information

Supplementary Material 1.

Supplementary Material 2.

Supplementary Material 3.

## Data Availability

Sequence data were submitted to NCBI GenBank (https://www.ncbi.nlm.nih.gov/) and can be found under the Project code PRJNA1322391 (https://www.ncbi.nlm.nih.gov/sra/?term=PRJNA1322391). All scripts and processed data are hosted on GitHub (https://github.com/biomicrodoc/Metania_microbiome_Alves-et-al.git). Metadata and results of statistical analyses are available in Additional files 1 and 2.

## Abbreviations

KEGG: Kyoto Encyclopedia of Genes and Genomes
KO: KEGG Ortholog
CRISPR: Clustered regularly interspaced short palindromic repeats
BGC: Biosynthetic gene clusters
BER: Base excision repair
DA: Differentially abundant
RiPP: Post-translationally modified peptides
GCF: Gene cluster families
ORF: Open read frames
DNA: Deoxyribonucleic acid
PERMANOVA: Permutational Multivariate Analysis of Variance
PCoA: Principal Coordinates Analysis
