# Supplementary material for: Taxonomic and functional diversity of the microbiome associated with the freshwater sponge Metania sp. (Haplosclerida: Metaniidae) from the Brazilian Cerrado, a metagenomic approach
Source: BMC Microbiol. 2026 Jun 20;26:563. doi: 10.1186/s12866-026-05301-3 (PMC13283328; doi:10.1186/s12866-026-05301-3)
Supplement: Supplementary file 3 — Supplementary Material 3. [file 12866_2026_5301_MOESM3_ESM.docx]

**Supplementary Material**

**Taxonomic and functional diversity of the microbiome associated with the freshwater sponge *Metania* sp. (Haplosclerida: *Metaniidae*) from the Brazilian Cerrado, a metagenomic approach**

**Author names: Carla Patrícia Pereira Alves^1^, Otávio Henrique B. Pinto^1,2,3^, Georgios Joannis Pappas Jr^2^, Sula Salani Mota^3^, Janina Rahlff ^4,5,6*^, Ricardo Henrique Krüger^1*^.**

**Authors E-mail:** [carlaalves@unb.br](mailto:carlaalves@unb.br), [otaviohenriquebp9@gmail.com](mailto:otaviohenriquebp9@gmail.com), [gpappas@unb.br](mailto:gpappas@unb.br), [sula.salani@undf.edu.br](mailto:sula.salani@undf.edu.br), [janina.rahlff@lnu.se](mailto:janina.rahlff@lnu.se), [kruger@unb.br](mailto:kruger@unb.br)

**Affiliations:**

^1^ Molecular Biotechnology Centre, Universidade de Brasília (UnB), Brasília 70910-900, Brazil

^2^ Genomics for Climate Change Research Center, Universidade Estadual de Campinas, Campinas, 13083-875, SP, Brazil

³ Centro de Biologia Molecular e Engenharia Genética, Universidade Estadual de Campinas, 13083-875, SP, Brazil

^4^Department of Cell Biology, University of Brasilia, Brasilia 70910-900, DF, Brazil

^5^ Universidade do Distrito Federal (UnDF) Professor Amaury Maia Nunes, Brasília-DF, Brazil

^6^ Leibniz Institute on Aging - Fritz Lipmann Institute (FLI), Jena, Germany

^7^ Department of Biology and Environmental Science, Centre for Ecology and Evolution in Microbial Model Systems (EEMiS), Linnaeus University, Kalmar, Sweden

^8^ Aero-Aquatic Virus Research Group, Faculty of Mathematics and Computer Science, Friedrich Schiller University Jena, Jena, Germany

**Corresponding authors:**

Dr. Ricardo Henrique Krüger

Molecular Biotechnology Centre, Universidade de Brasília (UnB), Brasília 70910-900, Brazil

[kruger@unb.br](mailto:kruger@unb.br)

Dra. Janina Rahlff

Leibniz Institute on Aging - Fritz Lipmann Institute (FLI), Jena, Germany

[janina.rahlff@lnu.se](mailto:janina.rahlff@lnu.se)


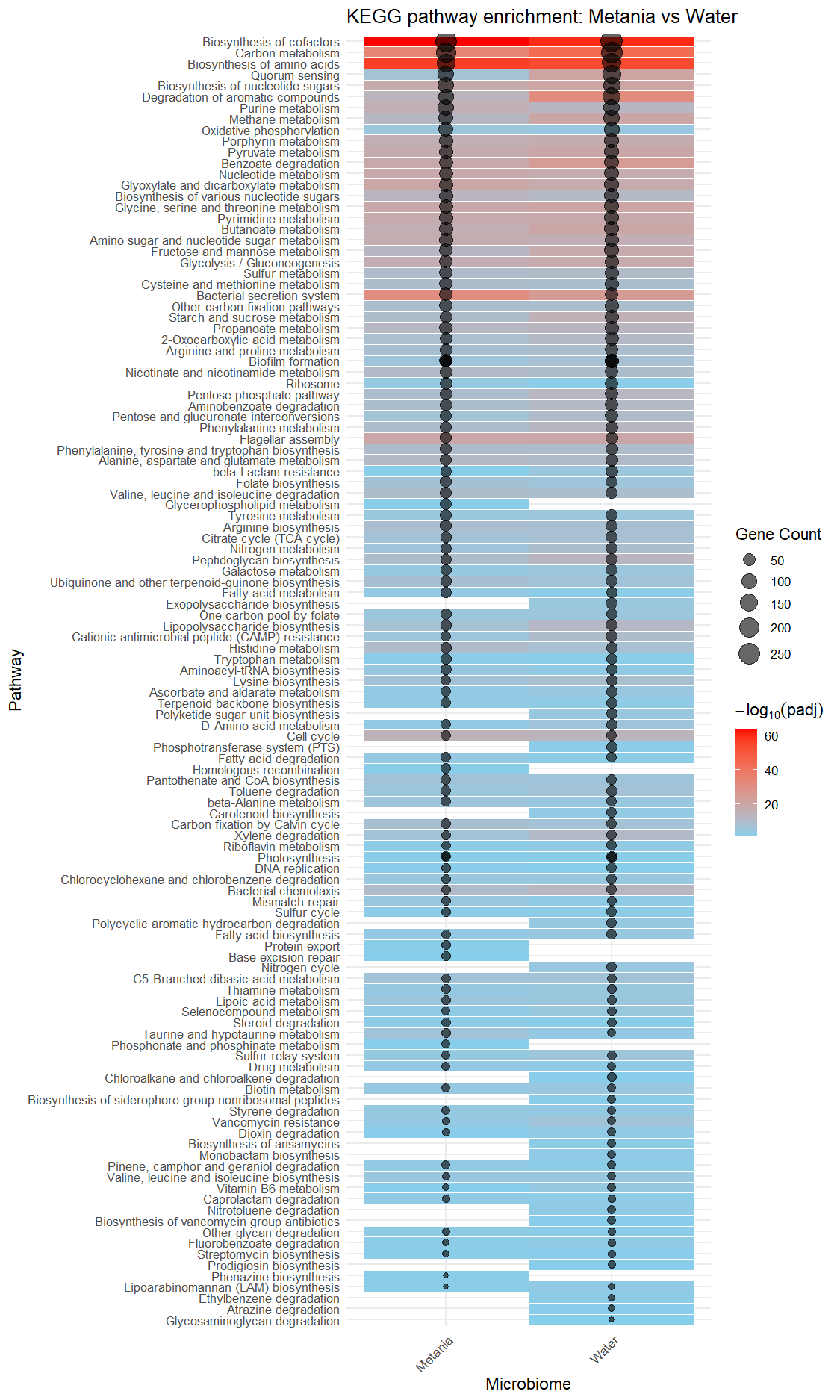


**Fig. S1** **Enrichment of KEGG pathways of the KOs in the *Metania* sp.- and surrounding water-associated microbiomes.** Bubble size represents the count of orthologous genes. Colors represent log p-adj, where red is most significant and blue is least significant.

**Table S1** **Biosynthetic gene clusters (BGCs) predicted by antiSMASH and matches BGC types in MIBIG repository**.

| **Microbiome** | **Type** | **MIBiG_ID** | **Description** | **Similarity (%)** |
| --- | --- | --- | --- | --- |
| ***Metania* sp.** | terpene | BGC0000637.3 | carotenoid | 37 |
|  | terpene | BGC0000647.3 | carotenoid | 100 |
|  | azole-containing-RiPP | BGC0002653.2 | macolacin | 40 |
|  | terpene | BGC0000647.3 | carotenoid | 100 |
|  | terpene | BGC0000647.3 | carotenoid | 100 |
|  | terpene-precursor | BGC0001756.3 | retigeranin/arathanatriene | 42 |
|  | azole-containing-RiPP | BGC0002653.2 | macolacin | 40 |
|  | terpene-precursor | BGC0001756.3 | retigeranin/arathanatriene | 42 |
|  | terpene | BGC0000637.3 | carotenoid | 37 |
|  | terpene | BGC0000647.3 | carotenoid | 100 |
|  | terpene | BGC0000647.3 | carotenoid | 100 |
|  | azole-containing-RiPP | BGC0000400.5 | paenibacterin | 40 |
|  | terpene | BGC0000637.3 | carotenoid | 37 |
|  | terpene | BGC0000647.3 | carotenoid | 100 |
|  | terpene-precursor | BGC0001756.3 | retigeranin/arathanatriene | 42 |
|  | terpene | BGC0000647.3 | carotenoid | 100 |
|  | terpene | BGC0000647.3 | carotenoid | 100 |
|  | terpene | BGC0000647.3 | carotenoid | 100 |
|  | terpene-precursor | BGC0001756.3 | retigeranin/arathanatriene | 42 |
| **Water** | terpene | BGC0000656.5 | zeaxanthin | 66 |
|  | terpene | BGC0000647.3 | carotenoid | 100 |
|  | terpene | BGC0000647.3 | carotenoid | 100 |
|  | terpene | BGC0000643.3 | carotenoid | 30 |
|  | terpene | BGC0000647.3 | carotenoid | 100 |
|  | terpene | BGC0000644.3 | carotenoid | 33 |
|  | terpene | BGC0000647.3 | carotenoid | 100 |
|  | terpene | BGC0000644.3 | carotenoid | 33 |
|  | terpene | BGC0000650.3 | carotenoid | 42 |
|  | terpene | BGC0000650.3 | carotenoid | 42 |
|  | terpene | BGC0000637.3 | carotenoid | 37 |
|  | terpene | BGC0000647.3 | carotenoid | 100 |
|  | terpene | BGC0000644.3 | carotenoid | 33 |
|  | terpene-precursor | BGC0001756.3 | retigeranin/arathanatriene | 42 |
|  | terpene | BGC0000644.3 | carotenoid | 33 |
|  | terpene | BGC0000637.3 | carotenoid | 37 |
|  | terpene | BGC0000644.3 | carotenoid | 33 |
|  | terpene | BGC0000637.3 | carotenoid | 37 |
|  | terpene | BGC0000647.3 | carotenoid | 100 |
|  | terpene | BGC0000644.3 | carotenoid | 33 |
|  | terpene | BGC0000650.3 | carotenoid | 42 |
|  | terpene | BGC0000643.3 | carotenoid | 30 |
|  | terpene | BGC0000647.3 | carotenoid | 100 |
|  | terpene | BGC0000643.3 | carotenoid | 30 |
|  | terpene | BGC0000647.3 | carotenoid | 100 |
|  | terpene | BGC0000650.3 | carotenoid | 42 |
|  | terpene | BGC0000644.3 | carotenoid | 33 |
|  | terpene | BGC0000647.3 | carotenoid | 100 |
|  | terpene | BGC0000647.3 | carotenoid | 100 |
|  | NRPS | BGC0001758.4 | rhizomide A/rhizomide B/rhizomide C | 100 |
|  | terpene-precursor | BGC0000866.5 | polyhydroxyalkanoate | 50 |
|  | terpene | BGC0000647.3 | carotenoid | 100 |
|  | terpene | BGC0000647.3 | carotenoid | 100 |
|  | terpene | BGC0000644.3 | carotenoid | 33 |
|  | terpene | BGC0000647.3 | carotenoid | 100 |
|  | terpene | BGC0000647.3 | carotenoid | 100 |
|  | terpene | BGC0000650.3 | carotenoid | 42 |
|  | terpene | BGC0000647.3 | carotenoid | 100 |
|  | terpene | BGC0000647.3 | carotenoid | 100 |
|  | terpene | BGC0000656.5 | zeaxanthin | 100 |
|  | terpene | BGC0000644.3 | carotenoid | 33 |
|  | terpene | BGC0000644.3 | carotenoid | 33 |
|  | terpene | BGC0000644.3 | carotenoid | 33 |
|  | terpene | BGC0000644.3 | carotenoid | 33 |
|  | terpene | BGC0000644.3 | carotenoid | 33 |
|  | terpene | BGC0000647.3 | carotenoid | 100 |
|  | terpene | BGC0000650.3 | carotenoid | 42 |
|  | terpene | BGC0000647.3 | carotenoid | 100 |
|  | terpene | BGC0000643.3 | carotenoid | 30 |
|  | terpene | BGC0000644.3 | carotenoid | 33 |
|  | terpene | BGC0000647.3 | carotenoid | 100 |
|  | terpene | BGC0000637.3 | carotenoid | 37 |
|  | terpene | BGC0000647.3 | carotenoid | 100 |
|  | terpene | BGC0000647.3 | carotenoid | 100 |
|  | terpene | BGC0000656.5 | zeaxanthin | 100 |
|  | terpene | BGC0000647.3 | carotenoid | 100 |
|  | terpene | BGC0000644.3 | carotenoid | 33 |
|  | terpene | BGC0000650.3 | carotenoid | 42 |
|  | terpene | BGC0000637.3 | carotenoid | 37 |
|  | terpene | BGC0000637.3 | carotenoid | 37 |
|  | terpene | BGC0000643.3 | carotenoid | 30 |
|  | terpene | BGC0000647.3 | carotenoid | 100 |
|  | terpene | BGC0000647.3 | carotenoid | 100 |
|  | terpene | BGC0000650.3 | carotenoid | 42 |
|  | terpene | BGC0000644.3 | carotenoid | 33 |
|  | terpene | BGC0000644.3 | carotenoid | 33 |
|  | terpene | BGC0000647.3 | carotenoid | 100 |
|  | terpene | BGC0001248.3 | clavaric acid | 100 |
|  | terpene-precursor | BGC0001756.3 | retigeranin/arathanatriene | 42 |
|  | terpene | BGC0000647.3 | carotenoid | 100 |
|  | terpene | BGC0000644.3 | carotenoid | 33 |
|  | NRPS-like | BGC0001822.4 | vioprolide A/vioprolide B/vioprolide C/vioprolide D | 33 |
|  | NRPS-like | BGC0001833.4 | icosalide A/icosalide B | 100 |

The columns show the microbiomes origin, the BGCs predicted by Antismash (type) MIBIG_ID, description, and similarity score (%). Here, we show the hits of low (30-75%) and high similarity (>75%).
